# Assessment of LED fluorescence microscopy for the diagnosis of *Plasmodium falciparum *infections in Gabon

**DOI:** 10.1186/1475-2875-10-194

**Published:** 2011-07-18

**Authors:** Dominic Lenz, Peter G Kremsner, Bertrand Lell, Barbara Biallas, Michael Boettcher, Benjamin Mordmüller, Ayola A Adegnika

**Affiliations:** 1 Medical Research Unit, Albert Schweitzer Hospital, Lambaréné, Gabon; 2 Institute of Tropical Medicine, University of Tübingen, Tübingen, Germany; 3 Department of Parasitology, Leiden University Medical Center, Leiden, The Netherlands

## Abstract

**Background:**

Rapid and accurate diagnosis of malaria is central to clinical management and the prevention of drug-overuse, which may lead to resistance development, toxicity and economic losses. So far, light microscopy (LM) of Giemsa-stained thick blood smears is the gold standard. Under optimal conditions the procedure is fast and reliable; nevertheless a gain in speed would be a great advantage. Rapid diagnosis tests are an alternative, although they cost more and give qualitative instead of quantitative results. Light-emitting diode (LED) fluorescence microscopy (ledFM 400 ×, 1000 ×) may offer a reliable and cheap alternative, which can be used at the point of care.

**Methods:**

LedFM and conventional fluorescence microscopy (uvFM) were compared to LM in 210 samples from patients with history of fever in the last 24 hours admitted to the Albert Schweitzer Hospital in Lambaréné, Gabon.

**Results:**

Sensitivities were 99.1% for ledFM and 97.0% for uvFM, specificities 90.7% for ledFM 400 × and 92.6% for ledFM 1000 × and uvFM. High agreement was found in Bland-Altman-plot and Kappa coefficient (ledFM 1000 ×: 0.914, ledFM 400 × and uvFM: 0.895). The time to diagnosis for both FM methods was shorter compared to LM (LM: 43 min, uvFM: 16 min, ledFM 1000 ×: 14 min, ledFM 400 ×: 10 min).

**Conclusion:**

ledFM is a reliable, accurate, fast and inexpensive tool for daily routine malaria diagnosis and may be used as a point of care diagnostic tool.

## Background

In 2010, malaria is still endemic in more than 100 countries with 2.2 billions of people at risk. This results in 300-500 million clinical episodes and more than one million deaths with a 90% burden in sub-Saharan African countries [1]. As a major health problem, malaria unfortunately is still lacking a rapid and accurate diagnostic tool. Thick blood smears stained in Giemsa and examined with light microscopy are a gold standard method. However, this is time consuming, demands experienced technicians, and requires proper preparation and replacement of the dye at least 2-3 times per day to maintain precise results. In practice, these requirements are rarely fulfilled leading to a lack of accurate diagnosis, which results in presumptive treatment. In times of decreasing incidence and prevalence, as well as lower parasitaemia, but rapid emergence of resistance and expensive drugs, new fast, easy and reliable tools for malaria diagnosis are required. Rapid diagnosis tests (RDT), are fast and reliable, but only give qualitative results. In addition, they are comparatively expensive and have a short shelf life. Therefore RDTs are not an ideal diagnostic tool for the primary-care level [2].

An alternative technique is fluorescence microscopy based on light-emitting diodes (LED) of one wavelength using acridine orange as a nucleic acid fluorescent dye, which stains DNA and RNA instantly. Such microscopes were recently approved for fast Tuberculosis diagnosis using an auramine-rhodamine dye [3]. This is a very useful tool in field-settings as the LED consume less energy, are long-lasting and brighter, as a result of which they do not require darkrooms; these have been major drawbacks of conventional fluorescence microscopy. Additionally, they offer battery operation during power shutdowns or in areas where no electricity is available allowing fast and accurate diagnosis even under these circumstances. Previous studies have already shown the use of acridine orange in malaria diagnosis using conventional fluorescence microscopy or an interference filter system [4-6]. But this was not considered a useful tool in field conditions due to weak illumination or high costs. In the following study, conventional light microscopy (LM) of Giemsa-stained thick blood smears were compared to the new LED fluorescence technique (ledFM) and conventional fluorescence microscopy (uvFM).

## Methods

The study was approved by the local ethics committee of Lambaréné (Comité d'Éthique Régional Indépendent de Lambaréné) and carried out between September and November 2009 at the Albert Schweitzer Hospital, Lambaréné, Gabon (ASH) - an area of perennial malaria transmission. Blood samples of 210 anonymous patients from the outpatient department of the ASH with a history of fever within the last 24 hours and suspected diagnosis of malaria were included. From each participant, 1 ml of blood was collected in an ethylenediaminetetraacetic acid (EDTA) tube, the white blood cell count determined (ABX Micros 60OT, ABX Diagnostics, France) and transported directly to the laboratory for processing by the different methods: i) Giemsa-stained thick blood films, and wet mounts of acridine orange-stained blood examined under a ii) Nikon Optiphot-2 mercury lamp epifluorescence microscope (filter: B2A, excitation maximum: 470 nm) at 1,000 × magnification and under a Zeiss Primo Star iLED epifluorescence microscope equipped with a 455 nm LED at iii) 400 × and iv) 1,000 × magnification. The Giemsa-stained thick film was prepared and read at a 1,000 × magnification according to WHO guidelines [7]. For the other three techniques, 8 μl of EDTA-anticoagulated blood was incubated in a 0.5 ml reaction tube with 2 μl of acridine orange solution (1 mg/ml in PBS, pH 7.4, weekly prepared and stored in the dark) for three minutes at room temperature. Subsequently, blood cells were applied as a wet mount onto a slide, covered with a cover slip and closed with Eukitt (Sigma, Germany).

For all methods parasitaemia was assessed by counting white blood cells and parasites until 200 white blood cells (WBC) were observed; if more than 10 parasites were found, the parasitaemia was calculated as shown below. Otherwise counting was continued until 500 WBC. If no parasite was found, the slide was declared negative. The following equation was used to calculate the parasitaemia per microlitre:

For each method, the mean of the two results of two independent readers was taken as the final result. In case of a > 2-fold disagreement in the number of parasites, the reading was repeated by a third independent reader. The mean of the two closest results of the three readings was taken as the final result. Reading time was defined as the mean time of two readers from the beginning of microscopic examination to the reporting of the parasitaemia. Turnover time was defined as reading time plus time of preparation from check-in of the blood sample to microscopy. Time of detection of the first parasite was defined as time from start of microscopy to detection of the first parasite in the sample.

To assess inter-observer agreement, ten different readers read two sets of three slides of low, middle, and high parasitaemia. The first set was read by ten technicians of the laboratory in LM, the second set by another ten technicians in ledFM (1,000 ×) after a short introduction lasting 30 min. Only three out of ten readers had ever used a fluorescence microscope before.

LM was chosen as the Gold Standard technique (GS). Data were entered into paper case report forms and then into an electronic database. Analyses were done with SPSS 16.0 for Windows (SPSS Inc., Chicago, USA). Agreement was measured using Cohen's Kappa test. Bland-Altman plots were used to compare graphically the agreement of two methods: data from parasitaemia was log_10 _transformed. The mean of log_10 _transformed parasitaemia from the final result was used to compare counts of one fluorescence method with the LM as the GS. Kaplan Meier plot and log rank test were done to compare the time to detect the first parasite in the positive samples. All microscopists read their set of slides independently and were blinded from the results of the other readers.

## Results

From 210 samples, 102 (LM), 107 (uvFM), 109 (ledFM, 1,000 ×) and 111 (ledFM, 400 ×) were rated positive. Analysis of sensitivity and specificity as illustrated in Table 1 showed sensitivities between 97 and 99% - with best values of 99% for both magnifications of ledFM. Specificity of the methods ranged from 91% for the 400 × ledFM up to 93% for the two other methods. The predictive values were assessed once according to the study population as a pre-selected population and a second time corrected for the current (2007 - 2008) prevalence of malaria among patients admitted to the ASH with history of fever [8].

**Table 1:** Sensitivity, specificity and predictive values

	LM	uvFM	ledFM × 400	Led FM × 1000
**false positive**	0	8	10	8
**true positive**	102	99	101	101
**false negative**	0	3	1	1
**true negative**	108	100	98	100

	210	210	210	210
				
**Sensitivity**	GS	97.1%	99.0%	99.0%
**Specificity**	GS	92.6%	90.7%	92.6%
				
**PPV**		92.7%	91.2%	92.8%
**NPV**		97.0%	99.0%	99.0%
				
***PPV cor.***		*49.7%*	*44.6%*	*50.2%*
***NPV cor.***		*99.8%*	*99.9%*	*99.9%*

				
**3^rd ^Readings**	12.4%	22.9%	14.8%	12.9%

Sensitivity, specificity and predictive values with LM as the Gold Standard are shown for ledFM and uvFM The PPV (positive predictive value) and NPV (negative predictive value) concern the study population and the corrected values used the prevalence of patients at Albert-Schweitzer Hospital with history of fever [8].

The agreement of the three fluorescence methods was assessed using the Cohen's Kappa test for agreement compared to the results of LM; all technique had similar results with a coefficient of 0.895 for uvFM and ledFM (400 ×) and of 0.914 for ledFM (1,000 ×). As a comparison, the inter-reader agreement of LM was likewise assessed and showed a similar result with a kappa coefficient of 0.904.

### Method comparison

**Figure 1:**
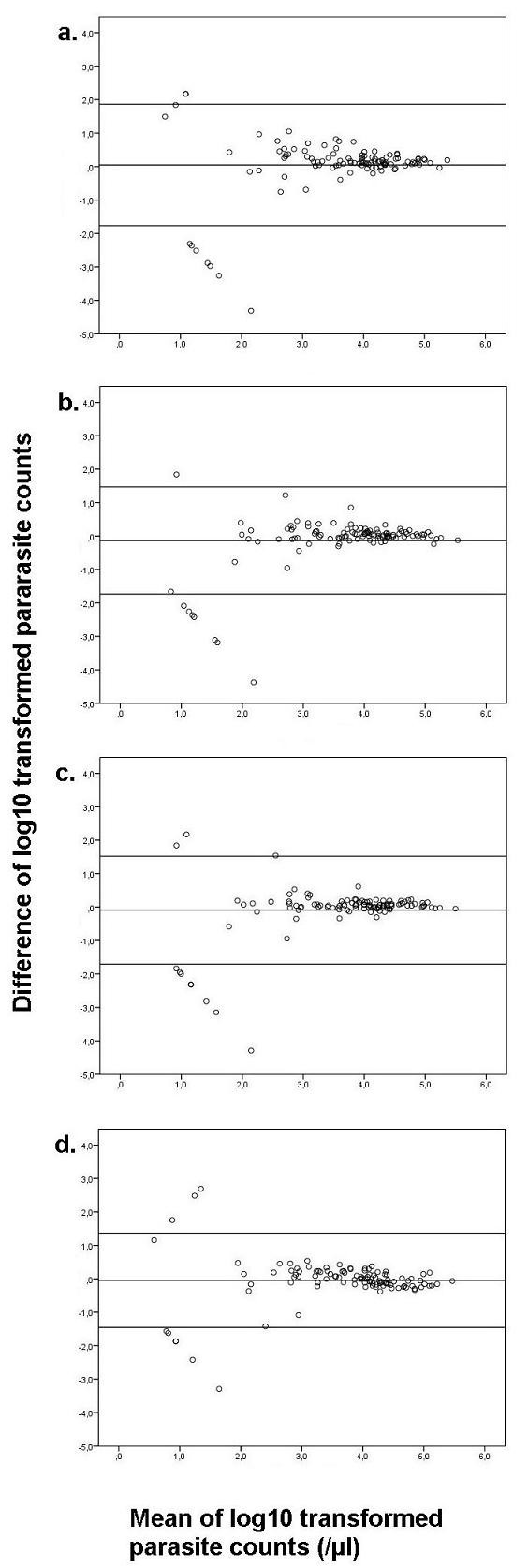
**Mean of log_10 _transformed parasite counts**. Bland-Altman plot of log10-transformed parasitaemia data showing difference means and upper and lower 95% limits of agreement. (a) Agreement of LM and uvFM. (b) Agreement of LM and ledFM (400 ×). (c) Agreement of LM and ledFM (1,000 ×). (d) Inter-observer-agreement within LM. **Note: **The divergent results outside the 95%-LOA are due to discordant readings between the methods except one in figure 1c. They are all occurring in low parasitaemia.

Bland-Altman plots showed almost no mean difference between the different fluorescent methods (Figure 1) and LM. To compare parasitaemia, the counts were first log_10_-transformed. The difference of the means (95% limits of agreement [LOA]) for uvFM and LM was 0.0478 (-1.77, 1.86, n = 103) (Figure 1a), for ledFM (400 ×) and LM -0.134 (-1.73, 1.47, n = 103) (Figure 1b) and for ledFM (1000 ×) and LM -0.092 (-1.70, 1.52, n = 103) (Figure 1c).

Additionally, the mean difference of single LM-readings was compared to assess concordance of microscopists. It showed a mean difference (95% LOA) of -0.045 (-1.45, 1.36, n = 103)(Figure 1d), which is similar to the comparisons between the methods. All results outside the 95% LOA except one in ledFM (1,000 ×) were due to discordant results regarding positivity of the two methods or readers.

### Inter-observer agreement

**Figure 2:**
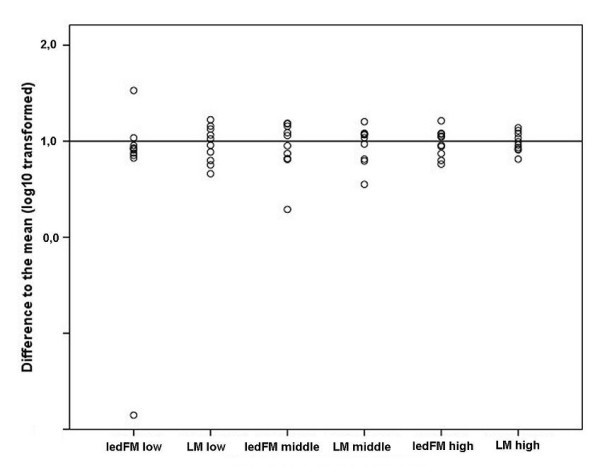
**Inter-observer agreement**. Log_10 _transformed difference to the mean result normalized as "1" of slide samples with low, middle and high parasitaemia in LM and ledFM (1,000 ×) read by 10 different readers.

Results of the readings were normalized and expressed as the confidence interval around one (Figure 2). For the normalization the mean of all readings of a slide was set as "1" and then plotted the log_10 _transformed difference of each reading to the mean. One microscopist did not detect low parasitaemia under ledFM. For LM, 95% confidence intervals (CI) were 0.83 - 1.16 for a high (20,800 parasites per μl), 0.74 - 1.3 for a medium (1,580 parasites per μl) and 0.71 - 1.29 for a low (635 parasites per μl) parasitaemia. Microscopy under ledFM led to CI of 0.77 - 1.2 for a high (31,790 parasites per μl), 0.68 - 1.3 for a medium (8,314 parasites per μl) and 0.37 - 1.6 for a low (712 parasites per μl) parasitaemia.

### Time to the result and detection of the first parasite

**Figure 3:**
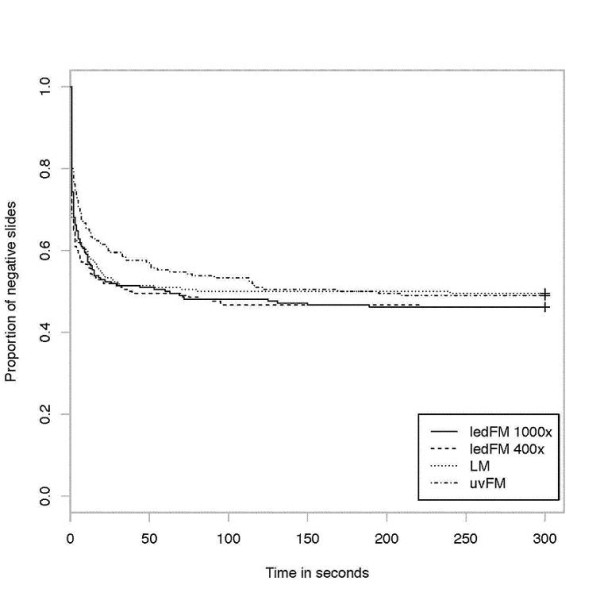
**Parasite detection time**. Kaplan-Meier-plot to show time until detection of the first parasite (truncated at 5 min).

Turn-over-time (min:sec) was shorter mainly due to short preparation time (Giemsa 37:02; acridine orange 4:37). The reading and turn-over-times (min:sec) were 5:20 and 42:22 for LM, 11:25 and 16:02 for uvFM, 5:20 and 9:57 for ledFM (400 ×) and 8:54 and 13:31 for ledFM (1000 ×). The comparison of the detection time using Kaplan-Meier-plot and log rank Test showed no significant differences (p = 0.67) between the methods (Figure 3).

### Third readings

Third readings, due to discordant results of the first two microscopic readings, were necessary in 12.4% (LM), 22.9% (uvFM), 14.8% (ledFM; 400 ×), and 12.9% (ledFM; 1,000 ×) of diagnoses (Table 1).

## Discussion

The results demonstrate a high sensitivity and acceptable specificity for the fluorescent techniques. The lower specificity may be at least partially due to missed positive samples in the gold standard compared to the acridine orange technique. This is not surprising since acridine orange was reported to be more sensitive than Giemsa staining elsewhere [5]. Agreement of ledFM with LM was better performing than that of WHO approved "conventional" uvFM [9] and is comparable to inter-observer agreement within the gold standard method. Similar results were obtained through visualization in Bland-Altman-plots.

In reference to the Inter-observer agreement, the wide confidence interval for low parasitaemia using ledFM was due to missed diagnosis of one reader. Exclusion of the non-performer leads to a CI of 0.41 - 1.59. This divergent result, the wider confidence intervals as well as the higher percentage of third readings may be explained by the short training of 30 min and the lack of readers experienced in fluorescent microscopy. However, the turn over time was improved largely - diagnosis was four times quicker using the ledFM (400 ×).

Due to the bright illumination of the specimen, ledFM can be used in normal daylight settings making the expensive installation of a darkroom unnecessary and the method valuable for emergency and primary care settings. The weak illumination reported using Kawamoto's interference filter system [6,10] was improved and therefore higher blood concentration can be examined reducing the time for parasitaemia assessment.

A potential disadvantage is that differentiation of Plasmodium species is more difficult in fluorescent specimen, although some mixed infections were occasionally recognized using ledFM when used in a routine setting in Lambaréné. Furthermore, a long-time storage of stained slides is not possible [11] as after a few hours the readers reported a fading of the staining which exclude further reading. This may not be important for the daily laboratory routine, but difficult in research settings where archiving of slides is important. However, previous studies reported a possible restaining with Giemsa for storage [12].

Other methods to diagnose malaria such as immunofluorescence antibody assays, enzyme-linked immunosorbent assays, or polymerase chain reactions (PCR) are time-consuming and expensive. Nevertheless, they have important roles in research, epidemiology, and screening of blood products. RDTs are an alternative fast and reliable diagnostic. But they give only qualitative results, have short shelf lives, and require temperature and humidity controlled storage, making the supply in remote areas difficult. Moreover, previous studies reported positivity of some tests after completed treatment due to the long half-live of the plasmodial protein that is detected by the assay [13]. Beside that, compared to microscopy, RDT are expensive (U.S. $0.55-$1.50 compared to U.S. $0.12-$0.40 for microscopy [2]). Since purchase of the Primo Star iLED is subsided by the Foundation for Innovative New Diagnostics (FIND) it is comparable to a conventional light microscope. Running costs are lower and purchase logistics are simpler since acridine orange is cheap and not inflammable.

## Conclusion

Acridine orange is a very fast stain with a long shelf-life stored as a powder in the dark. The solution for daily use is easy to prepare and can be kept throughout the week, whereas Giemsa staining is more elaborate to handle requiring frequent preparation, high experience, infrastructure and quality assurance making it time-consuming. The feasibility and performance of the acridine orange method in field settings has previously been shown in Tanzania using the interference filter system [14]. LED fluorescent microscopes recently became available and provide major advantages as they are long-lasting, less power consuming and can be used in daylight settings. The possibility of using a battery package during power shutdowns is an advantage that makes them even more suitable for daily routine especially in rural areas. In conclusion, ledFM is a cheap, accurate and, therefore, a very interesting tool for malaria diagnosis in field settings all over Africa.

## Conflict of interests by the authors

The authors declare that they have no competing interests.

## Authors' contributions

DL, PGK, BL, MB, BM and AA designed experiments; DL and BB performed experiments and collected the data; DL, BM and AA analysed data and wrote the manuscript; BL and PGK gave technical support and conceptual advice. All authors read and approved the final manuscript.
